# Global Fight against Malaria: Goals and Achievements 1900–2022

**DOI:** 10.3390/jcm13195680

**Published:** 2024-09-24

**Authors:** Marc Thellier, Ayawovi Arlene Jessicka Gemegah, Ilhame Tantaoui

**Affiliations:** 1 AP-HP, Centre National de Référence du Paludisme, Hôpital Pitié–Salpêtrière, 75013 Paris, France; ayawoviarlenejessicka.gemegah@aphp.fr (A.A.J.G.); ilhame.tantaoui@aphp.fr (I.T.); 2 Sorbonne Université, Institut National de la Santé et de la Recherche Médicale (INSERM), IPLESP Institut Pierre–Louis d’Épidémiologie et de Santé Publique, 75013 Paris, France

**Keywords:** malaria, *Plasmodium*, *Anopheles*, control, elimination, eradication, World Health Organization (WHO)

## Abstract

This article examines the historical and ongoing efforts to fight malaria, a parasitic disease caused by *Plasmodium* species and transmitted by *Anopheles* mosquitoes. Despite over a century of control efforts, malaria remains a major global health issue. In 2022, there were an estimated 249 million cases across 85 countries, leading to approximately 600,000 deaths. In the recently published Global Technical Strategy for Malaria 2016–2030, the World Health Organization (WHO) has prioritized malaria eradication. The main goals are to reduce malaria incidence and mortality by 90% by 2030 compared to 2015 levels. However, as of 2022, progress has been limited, with only a 2% reduction in incidence and a 6% reduction in mortality. This review traces the historical context of malaria, highlighting its ancient origins and the pivotal scientific discoveries in the late 19th century that paved the way for modern control measures. The Global Malaria Eradication Programme launched by the WHO in 1955 initially showed promise, largely due to the insecticide DDT, but ultimately failed to achieve its goals mainly due to logistical problems, vector resistance to DDT, and inadequate funding. Despite significant advances in the early 21st century, including the Roll Back Malaria initiative and increased international funding, malaria eradication remains a distant goal. Persistent challenges, such as weak healthcare systems, parasite and vector resistance to drugs and insecticides, and inadequate funding, continue to hamper global efforts. Therefore, this article underscores the need for a deeper understanding of malaria’s history and recent evolution to inform future strategies for eradication.

## 1. Present Situation, Extent of the Disease in the Year 2022

Malaria is an infectious parasitic disease caused by *Apicomplexan protozoa* of the genus *Plasmodium*. It is transmitted to humans by the bite of female mosquitoes of the genus *Anopheles* [1]. Among more than 150 species of *Plasmodium*, six are regularly responsible for malaria in humans: *Plasmodium falciparum*, *Plasmodium vivax*, *Plasmodium ovale curtisii*, *Plasmodium ovale wallikeri*, *Plasmodium malariae*, and *Plasmodium knowlesi* [1,2,3]. Due to its widespread geographic distribution and significant impact on human health, malaria remains one of the leading causes of morbidity and mortality worldwide. In the 20th century alone, malaria is estimated to have caused between 150 and 300 million deaths, accounting for 2% to 5% of all deaths [4]. In 2021, it ranked 14th among all causes of death and 4th in Africa [5]. In 2022, the number of malaria cases worldwide was estimated at 249 million across 85 countries and territories, resulting in approximately 600,000 deaths [6]. The WHO estimates that 77% of all deaths in 2022 were among children under five years of age. *Plasmodium falciparum* is responsible for more than 90% of malaria cases and deaths [6].

Malaria remains a significant concern for health authorities, who have identified it as one of the three diseases targeted for eradication by the WHO, alongside tuberculosis and HIV [7]. In its Global Technical Strategy for Malaria 2016–2030, the WHO aims to reduce malaria incidence and mortality by 90% between 2016 and 2030, compared with the baseline year of 2015, and to eliminate malaria in 35 countries, ensuring no resurgence in countries where it has been eliminated. The 2020 and 2025 milestones are to reduce malaria incidence and mortality by 40% and 75% and to eliminate malaria in at least 10 and 20 countries, respectively [8]. To achieve these goals, the WHO relies on a clear strategy, a network of proven partners, and a solid financial base, largely supported by the Global Fund, which accounted for 65% of global funding in 2022.

However, this strategy appears to be ineffective. In 2020, only one of the three intermediate targets of the milestone was on track: six of the ten countries targeted had achieved the goal of zero indigenous malaria cases for at least three consecutive years. The other two targets, to significantly reduce incidence and mortality, were far from being met. It is also highly unlikely that the 2025 intermediate milestone will be met or even approached [9]. Between 2015 and 2022, malaria incidence fell by only 2%, from 59.8 to 58.4 per 1000 of the population at risk, and the mortality rate decreased by only 6%, from 15.2 to 14.3 per 100,000 of the population [6]. In absolute terms, the total number of malaria cases actually increased by almost 8%, from 231 million in 2015 to 249 million in 2022 [6]. While the COVID-19 pandemic likely exacerbated the situation by disrupting malaria control efforts, it alone cannot explain these disappointing results. Negative trends were already evident between 2016 and 2019, and these trends continued post-pandemic in 2021 and 2022 [6]. Projections for 2030, if current trends continue, are even more alarming: incidence is expected to be 54.5 per 1000 people, and the mortality rate is expected to be 12.9 per 100,000 people, compared with targets of 6.0 and 1.5, respectively [6].

These figures demonstrate the resilience of malaria in the face of global eradication efforts. To fully understand the current and future challenges, it is critical to examine the origins of this disease and its impact long before the era of coordinated control efforts. Far from being a modern scourge, malaria has affected entire societies for thousands of years. Understanding its evolution, from the earliest historical records to the WHO era, is essential. By describing the major scientific discoveries and analyzing the successes and failures of malaria control programs throughout history, we can develop more effective strategies to achieve the ultimate goal: the eradication of malaria.

## 2. Malaria in the History of Mankind before 1900

**Figure 1:**
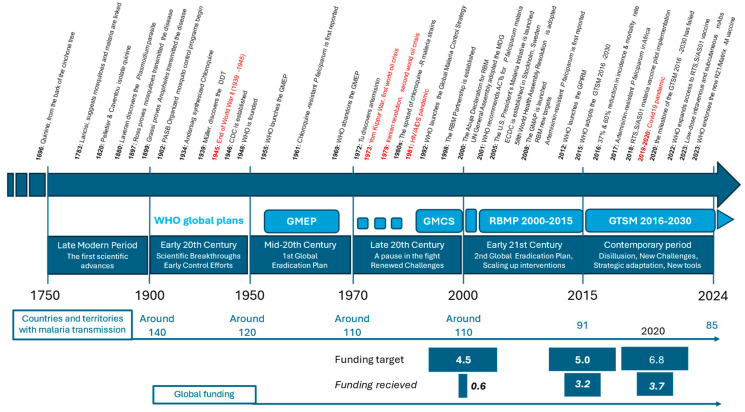
Timeline of the fight against malaria 1750–2024. PASB: Pan American Sanitary Bureau; DDT: dichlorodiphenyltrichloroethane; CDC: Centers for Disease Control and Prevention; WHO: World Health Organization; GMEP: Global Malaria Eradication Programme; RBM: Roll Back Malaria; UN: United Nations; MDG: Millenium Development Goals; ACTs: artemisinin combined therapies; ECDC: European Centre for Diseases Prevention and Control; WHA: World Health Assembly; GPIRM: Global Plan for Insecticide Resistance Management; RTS,S/AS01 and R21/Matrix-M: malaria vaccines both targeting the circumsporozoite antigen (CSP) using the RTS,S vector; mAbs: monoclonal antibodies. For more details, see Box 1.

Malaria, often referred to as “rhythmic swamp fever”, was described in ancient times across many parts of the world. Literary references to malaria date back to the earliest writings of human civilizations. The disease may have first been documented in China in around 2700 BC in the Nei Ching, or Canon of Internal Medicine, a treatise combining philosophy, medicine, and religion [10]. At that time, malaria was attributed to three demons responsible for headaches, chills, and fever. The disease also appears to have been described on clay tablets from 2000 BC in Mesopotamia and in Egyptian papyri and various ancient Egyptian monuments from 1600 to 1500 BC, where it is referred to as an illness characterized by fever, chills, and an enlarged spleen. This typical fever pattern was later mentioned in Indian writings from the Vedic period, between 1500 and 800 BC, where the disease was described as the “king of diseases” and attributed to the wrath of the god Shiva. In the 5th century BC, around 2500 years ago, Hippocrates, the father of Greek medicine, also described the rhythmic fever typical of malaria, which he associated with the appearance of Sirius, the Dog Star visible in late summer and autumn [11,12,13]. More recently, in the late modern period in 1717, Giovanni Maria Lancisi, an Italian scientist, suggested a link between mosquitoes and malaria. In his publication De Noxiis Paludum Effluviis (On the Noxious Effluvia of Marshes), he theorized that “poisonous vapors” from marshes mal’aria or “bad air” contributed to the disease and recommended draining the marshes around Rome (Figure 1). The presence of malaria pathogens in humans during these different times and places has recently been demonstrated by studying ancient parasite DNA (aDNA) in human remains [14,15]. Genetic analysis of aDNA, specifically looking for the *STEVOR*, *AMA1*, or *MSP1* genes specific to *P. falciparum*, revealed the presence of this *Plasmodium* species in the mummy of Tutankhamun and three other members of this lineage, dating from 3300 to 3400 years ago [16]. Infections with *P. falciparum* were common, as shown by several aDNA studies in Egypt, where about 20% of the subjects studied were infected [17,18]. In other samples, infections with the *P. vivax* species have been traced back almost 4000 years in Germany and Russia [14]. The presence of both *P. falciparum* and *P. vivax* has also been detected in human remains from the 6th century at the Versailles site in France [19].

Two fundamental discoveries at the end of the late modern period, at the end of the 19th century, paved the way for targeted malaria control (Figure 1). The first was the discovery of the parasite responsible for the disease in 1880 in Algeria by a French military doctor, Alphonse Laveran. He received the Nobel Prize in Physiology or Medicine in 1907, 27 years later, for his discovery. The second was the demonstration that malaria is transmitted by mosquito bites, made in 1895 in India by a British military doctor, Sir Ronald Ross, who was awarded the Nobel Prize in Physiology or Medicine in 1902 [20,21,22]. In 1899, Italian scientists Giuseppe Bastianelli, Amico Bignami, and Giovanni Battista Grassi proved that only mosquitoes of the genus *Anopheles* could transmit the disease [23]. The discoveries of malaria pathogens and vectors by French and English military doctors were no accident; they clearly illustrate the frequency and impact of malaria epidemics on conquering armies, particularly in highly exposed areas of Africa and Asia. The discovery of quinine, an alkaloid derived from the bark of the cinchona tree, first introduced to Europe from Peru at the end of the 17th century and later synthesized in 1820 by French chemists Pierre Joseph Pelletier and Joseph Bienaimé Caventou, greatly accelerated the expansion of European colonial empires across the globe [24,25,26].

From 1900 to the present day, the fight against malaria can be divided into two main phases: before and after the creation of the World Health Organization (WHO) in 1948 (Figure 1). The WHO was established in the aftermath of World War II as a result of the political will of 51 UN member states and 10 other countries to protect the health of their populations in an equal and coordinated manner [27].

Box 1 **Timeline of the fight against malaria: 1750–2024.**

■ **1696**: Quinine, derived from the bark of the cinchona tree, is first used to treat malaria, becoming the primary treatment in Europe and the Americas.
■ **1717**: Giovanni Maria Lancisi, an Italian scientist, suggests a link between mosquitoes and malaria, theorizing that “poisonous vapors” from marshes (mal’aria) or bad air contribute to the disease. He recommended draining the marshes around Rome.
■ **1740s**: The use of cinchona bark for treating “ague” (malaria) becomes widespread in Europe.

**Late Modern Period (1750–1899)**

■ **1820**: French chemists Pierre Joseph Pelletier and Joseph Bienaimé Caventou successfully isolate quinine from cinchona bark, making it the first alkaloid to be extracted for medicinal use. This breakthrough greatly enhances the effectiveness and availability of malaria treatment.
■ **1880**: Alphonse Laveran, a French Army doctor, discovers the *Plasmodium* parasite in the blood of a malaria patient in Algeria, identifying it as the cause of malaria. This work earns him the Nobel Prize in 1907.
■ **1897**: Sir Ronald Ross, a British Army doctor, proves that malaria is transmitted by mosquitoes, a discovery that earns him the Nobel Prize in 1902.
■ **1899**: Italian scientists Giuseppe Bastianelli, Amico Bignami, and Giovanni Battista Grassi proved that only mosquitoes of the genus *Anopheles* could transmit the disease.

**Early 20th Century (1900–1949): Scientific Breakthroughs and Early Control Efforts**

■ **1902**: Organized mosquito control programs begin, notably in the Panama Canal Zone, drastically reducing malaria cases.
■ **1934**: Chloroquine is synthesized by the scientist Johann Andersag at Bayer in Germany. It becomes widely used after World War II due to its effectiveness and low cost.
■ **1939**: Paul Hermann Müller, a Swiss chemist, discovers the insecticidal properties of DDT, leading to widespread mosquito control efforts. Müller receives the Nobel Prize in 1948.
■ **1946**: The U.S. Centers for Disease Control and Prevention (CDC) is established in Atlanta, Georgia, primarily to combat malaria in the United States.
■ **1948**: The World Health Organization (WHO) is founded, providing a global platform for coordinating health initiatives, including malaria control.

**Mid-20th Century (1950s–1960s): The Global Eradication Campaign**

■ **1951**: The U.S. National Malaria Eradication Program successfully eliminates malaria as a significant public health issue.
■ **1955**: WHO launches the Global Malaria Eradication Program (GMEP), aiming to eliminate malaria worldwide through extensive use of DDT and chloroquine distribution.
■ **1961**: Chloroquine-resistant *P. falciparum* is first reported in Southeast Asia and South America, complicating global malaria control efforts.
■ **1969**: WHO abandons the GMEP due to logistical challenges, increasing resistance to insecticides and drugs, and difficulties in sustaining the program in sub-Saharan Africa.

**Late 20th Century (1970s–1990s): Renewed Focus and Challenges**

■ **1972**: Chinese scientist Tu Youyou discovers artemisinin, derived from the sweet wormwood plant (*Artemisia annua*), which proves highly effective against malaria. This discovery lays the foundation for modern artemisinin-based therapies, and Tu Youyou was awarded the Nobel Prize in 2015.
■ **1973**: Artesunate, a derivative of artemisinin, is developed in China and becomes one of the most effective treatments for severe malaria.
■ **1980s**: The spread of chloroquine-resistant *P. falciparum* strains worsens the global malaria burden, particularly in Africa and Southeast Asia.
■ **1992**: WHO introduces the Global Malaria Control Strategy, shifting focus from eradication to control through improved diagnosis and treatment.
■ **1998**: The Roll Back Malaria (RBM) Partnership is established by WHO, UNICEF, UNDP, and the World Bank, aiming to halve malaria deaths by 2010 through coordinated global efforts.

**Early 21st Century (2000–2015): Scaling Up Interventions**

■ **2000**: The Abuja Declaration is signed by African leaders, committing to reducing malaria mortality by 50% by 2010. This period sees significant increases in funding and political commitment to malaria control.
■ **2001**: WHO recommends artemisinin-based combination therapies (ACTs) as the first-line treatment for *P. falciparum* malaria, replacing chloroquine.
■ **2005**: The U.S. President’s Malaria Initiative (PMI) is launched, boosting U.S. support for malaria control in Africa, including funding for insecticide-treated bed nets, indoor residual spraying, and ACTs.
■ **2005**: The European Centre for Disease Prevention and Control (ECDC) is established in Stockholm, Sweden.
■ **2008**: The Global Malaria Action Plan (GMAP) is launched by the RBM Partnership, with targets to reduce global malaria cases by 75% and eliminate malaria in 8–10 countries by 2015.
■ **2015**: WHO adopts the Global Technical Strategy for Malaria 2016-2030, aiming to reduce malaria incidence and mortality by at least 90% by 2030. The strategy emphasizes sustained investment, innovation, and expanding access to prevention and treatment.

**Contemporary History (2016–2023): Progress, Malaria Vaccines, New Challenges**

■ **2016**: the MDG indicators, Indicator 6.6 shows a 37% reduction in the incidence rate and a 60% reduction in the mortality rate by 2015 compared with 2000, preventing an estimated 6.8 million deaths, primarily among children under five in sub-Saharan Africa.
■ **2018**: The RTS,S/AS01 (Mosquirix) *P. falciparum* malaria vaccine, the first of its kind, begins pilot implementation in Ghana, Kenya, and Malawi. The vaccine is expected to complement existing malaria control measures.
■ **2022**: WHO expands access to the RTS,S/AS01 *P. falciparum* malaria vaccine, with plans to roll out the vaccine in more endemic countries in Africa.
■ **2022-2023**: Trials have shown the effectiveness of low-dose subcutaneous or intravenous monoclonal antibodies in preventing *P. falciparum* malaria.
■ **2023**: WHO endorses the new R21/Matrix-M *P. falciparum* malaria vaccine, providing an additional tool in the fight against malaria, particularly in regions with high transmission rates.

## 3. The Fight against Malaria before WHO Coordination: 1900–1950

The discovery of the malaria parasite and its life cycle at the end of the 19th century enabled scientists to map the geographical extent of the disease, revealing its presence far beyond the tropics. In Europe, for instance, malaria was widespread in Italy, particularly in the Pontine marshes and the Po Valley, as well as in the Iberian Peninsula (Spain and Portugal), Greece, Romania’s Danube Delta, and France’s Poitevin marshes, Camargue, and even in the Île-de-France region around Paris. It was also common in the Thames estuary in Great Britain, the coastal marshes of Flanders and the Netherlands, and as far north as the Baltic coast in Sweden [14,28,29]. By the early 20th century, populations on more than half of the Earth’s surface (53%) were at risk of malaria. Europe, Asia, the Americas, Oceania, and Africa were all affected by deadly malaria epidemics. Between 1900 and 1950, malaria was estimated to cause 2 to 3 million deaths per year worldwide, with the majority of these deaths occurring in densely populated tropical regions of Asia and Oceania [4,28].

At the dawn of the 20th century, malaria was recognized as a major public health problem and a significant obstacle to socio-economic development. It also became apparent that the knowledge of the parasite’s life cycle, combined with the discovery of quinine and the first insecticides, provided medicine and public health with effective tools to treat the disease and control or prevent its transmission. However, the fight against malaria was organized in vastly different ways, depending on the political and social context of each part of the world.

On the American continent, the Pan American Sanitary Bureau (PASB), founded in 1902, took an early lead in the fight against malaria (Figure 1). At its Third International Health Conference in 1907, PASB recommended that member governments include malaria in port health notifications, widely disseminate information about the disease, distribute quinine free of charge, and exempt all malaria prevention products from customs duties. In 1938, at its Tenth Conference, the PASB established the Pan-American Malaria Commission, tasked with studying the disease and developing comprehensive measures to combat it, including vector and parasite control, as well as relevant legislation [30]. In 1923, the League of Nations’ Hygiene Organization established the Malaria Commission, which focused on integrating malaria control into rural health programs [31].

The fight against malaria was not limited to international organizations. In many regions where the disease was endemic, colonial empires, primarily the British and French, were responsible for malaria control. Since the beginning of colonial conquests in Africa and Asia in the 1830s, colonial soldiers had died more frequently from a lack of prevention and effective treatment than from combat. Up to half of British and French soldiers died of mosquito-borne diseases before completing their first year in the tropics [32]. At the beginning of the 20th century, even with adequate quinine treatment, disease mortality remained high. In Madagascar, for example, of the 21,600 troops landed by France in 1895, 25 were killed in action, while within a few months, 5567 (26%) died of disease, mainly malaria [33]. So, initially, the governments of these empires relied heavily on military health services to study, diagnose, and treat tropical diseases among troops and expatriates. Later, in the first decades of the 20th century, these colonial powers began to extend protection to the general population, who were essential to the colonial economy. As a result, the population benefited from a health system with a functioning network and services to combat major endemic diseases. In the French territories and colonies, for example, nearly 4000 health facilities were established and maintained, including 41 major hospitals, 600 medical centers, 600 maternity wards, 350 leprosaria, and 2000 dispensaries, supported by training and research centers, including 15 Pasteur Institutes [34]. Interestingly, between 1900 and 1945, following the Industrial Revolution, the development of medicine and public health, along with improvements in living conditions, led to a significant decline in malaria in affected areas of Europe and North America. As an example, in the early 19th century, malaria was endemic throughout the United States, apart from northernmost New England, the mountains, and the deserts. It was hyperendemic in the swampy areas of the southern states, especially Alabama, Arkansas, South Carolina, Florida, Georgia, Louisiana, Mississippi, and Texas during the hot, humid months [35,36]. The species involved in disease transmission were *P. falciparum* and *P. vivax*. The malaria epidemic was closely linked to socio-economic and sanitary conditions. In the late 19th century, efforts were made to control malaria through a combination of local initiatives and public health campaigns [35]. However, in the 20th century, during the economic depression of the 1930s, both the disorganization of collective prevention (drainage projects were neglected or abandoned) and the lack of access to healthy living conditions or individual care contributed to the rapid resurgence of the disease throughout the country. At that time, the creation of the Tennessee Valley Authority (TVA) and its program of mosquito control through water management and swamp reclamation was one of the first major initiatives to combat malaria [37]. In the years that followed, economic, sociological, and public health programs were combined to reduce the incidence of malaria. However, by the outbreak of World War II, the risk of malaria in the United States was still significant in rural areas of the South where army fields and bases were located. In addition, American soldiers sent to endemic regions such as the South Pacific, North Africa, and Southeast Asia were exposed to malaria. Thus, the return of hundreds of thousands of potentially infected soldiers posed a major threat of disease resurgence in the United States [35,36]. In 1942, the Office of Malaria Control in War Areas (MCWA) was established to prevent malaria-infected soldiers from reintroducing the disease or reigniting epidemics in their home states. MCWA activities included insecticide spraying campaigns, swamp management and reclamation programs, and bite prevention education. Under the leadership of public health pioneer Dr. Joseph W. Mountin, a branch of the MCWA in Atlanta, Georgia, became the Communicable Disease Center (CDC) on 1 July 1946 (Figure 1). Shortly thereafter, it changed its name to the Centers for Disease Control and Prevention, with the same abbreviation [38]. The CDC, now a civilian organization with increased funding and dedicated human, scientific, and technical resources, continued these efforts. Most of its resources were devoted to spraying insecticides to eliminate disease-carrying mosquitoes. Thanks to these exemplary efforts, malaria was considered to be eliminated from the United States in 1951 [36].

In short, the drainage of marshlands, land reclamation, and improved water management, as well as the pollution of standing water (fertilisers, pesticides), reduced mosquito breeding sites, thereby decreasing their populations. Agricultural innovations have improved both human and animal health. Additionally, better construction practices and higher living standards have contributed to the reduction of malaria, particularly through the gradual separation of human and animal habitats, which has further also reduced transmission [28,39,40,41]. But, it is the targeted treatment of larval breeding sites, and particularly the crucial contribution of residual insecticides, that has made it possible to eliminate malaria in many developed countries.

However, despite some progress, malaria control was far less effective in the tropical regions of Africa, Asia, and South America, where the disease remained most prevalent. The establishment of accurate health networks, combined with the treatment of patients with quinine and the gradual development of these territories, was insufficient to effectively control malaria in these regions.

## 4. The Fight against Malaria under WHO Coordination: From 1951 to the Present Day

This second phase of the global fight against malaria was coordinated by the WHO. The organization was established on 7 April 1948 and formally began its work on 1 September 1948. Within a few years, the WHO was able to define the strategic goals of the global fight against malaria and organize and launch technical plans to implement them. This phase of malaria control can itself be divided into three periods (Figure 1). The first period corresponds to the Global Malaria Eradication Programme (GMEP), which aimed to achieve a malaria-free world. The GMEP was launched in 1955 and was officially abandoned in 1969. The second period, from 1970 to 1999, corresponds to the abandonment of the eradication goal, with a pause in the coordination of global efforts to combat the disease. Finally, the third period, after 2000, marked a return to the goal of eradication, with renewed determination to provide the political and financial resources necessary for both the strategic coordination and technical implementation of planned actions. The first technical plan was progressively implemented between 2000 and 2015, followed by the current plan for 2016–2030.

### 4.1. The First Coordinated Action between 1951 and 1969 

After the trauma of the Second World War, the nations of a rebuilding world decided that a global fight against the major scourges of humanity was both necessary and feasible. This fight would be coordinated by the newly created WHO. The stated goal for malaria, based on a long-term global program, was to eradicate the disease. Eradication (from the Latin eradicare, “to uproot”) involves the complete removal of the cause. Therefore, it should only be applied on a global scale, with no more malaria transmission in the world. The idea that malaria could be eradicated was supported by the remarkable effectiveness of an insecticide, dichlorodiphenyltrichloroethane (DDT), and an antimalarial drug, chloroquine, in the early 1950s, which made it possible to eliminate malaria in limited areas [30,42,43,44]. Moreover, spraying DDT inside houses was shown to be sufficient to interrupt malaria transmission, as demonstrated in Sardinia between 1946 and 1950, where the chemical was sprayed widely in the environment or in small amounts on the walls of houses [45]. At the end of the war, in the USA, the newly formed CDC, along with the TVA and the Rockefeller Foundation, began planning and funding the large-scale use of DDT as a residual insecticide on house walls and as a larvicide to control malaria. The results were spectacular: by 1951, malaria was eliminated, as compared to millions of cases just a few years earlier [46]. Elimination (from the Latin eliminare, “to put out, at the door”) involves a less radical notion of boundaries within which what is to be eliminated disappears. Eliminating a disease from an area can be seen as a necessary step towards the goal of eradication. Conversely, another option to fight the disease would be to control it by maintaining it permanently at a low level of transmission that is “tolerable” for human societies without attempting to suppress it completely. It should be noted that eliminating the disease in certain areas or countries may also be part of an overall disease eradication policy. In the case of malaria, however, control would be costly in human lives, with the ever-present risk of the epidemic returning to high levels of transmission [30,43].

### 4.2. The 1955 Turning Point and the Launch of the First Global Malaria Eradication Program

1955 marked a significant turning point in the fight against the disease, with the launch by the WHO of the first ambitious plan to eradicate the disease, the Global Malaria Eradication Programme (GMEP). This plan focused mainly on entomological control using DDT, a broad-spectrum, residual (long-lasting) insecticide capable of affecting malaria transmission by repelling or killing anopheline vectors [44]. To effectively target *Anopheles* mosquitoes before they come into contact with the human host, an insecticide sprayed on the interior walls of houses must be present at effective levels on a continuous basis [45,47]. Because it lasts for more than 6 months on indoor walls, DDT only needs to be applied twice a year. This greatly simplifies logistics and is less costly than products available at the time with shorter efficacy durations. When DDT is sprayed inside all the houses in an area just before the transmission period, it can greatly disrupt the dynamics of the vector population and significantly shorten the transmission periods. As expected, this campaign dramatically reduced the incidence and mortality of malaria wherever DDT was widely used. Some countries eliminated the disease, while others were close to doing so [48]. In India, for example, the number of cases fell from 75 million to a few thousand in a few years, and the mortality rate was close to zero in the early 1960s [49,50]. In just ten years, DDT saved millions of lives. According to the WHO, the global mortality rate fell by 90% between 1900 and the end of the 20th century [51].

### 4.3. Challenges and the End of the GMEP 

However, the effectiveness of DDT in malaria has been shown to be greatest in areas of unstable transmission, with pronounced seasonal peaks, rather than in areas of high and constant transmission. DDT was also being used intensively at the time to prevent insect damage to crops. This intensive use was not without consequences. DDT persisted in the environment for an extended period. Additionally, even though the toxic effects on humans are still debated, it was found to be more toxic than initially expected to both domestic and wild animals [50,52,53]. Resistance was also rapidly developed in certain *Anopheles* mosquito species that transmit the disease. Another factor that was not properly considered was the difficulty of applying this program in all situations. In fact, in the poorest countries with high malaria transmission, the different biological characteristics of the vectors, as well as challenges such as (i) a lack of public health personnel trained in insecticide spraying techniques, (ii) logistical issues for efficient and regular transport of equipment and personnel, (iii) extreme weather conditions (rain and floods, severe droughts), and finally, (iv) a lack of health education among the population with low adherence to control programs, all posed significant obstacles to the implementation of the eradication plan. In such situations, substantial external funding was essential if the goal was to be achieved. Unfortunately, the policies developed under the GMEP did not sufficiently consider the contributions of the countries and populations concerned. Only three independent countries in Sub-Saharan Africa represented at the 8th World Health Assembly in Mexico in 1955 (Ethiopia, Liberia, and South Africa) were able to partially benefit from this strategy [48]. As a result, strategic and technical decisions made at the global level were not, or only to a limited extent, aligned with local priorities and needs. Communication was inadequate, and funding was totally insufficient. Between 1956 and 1963, only USD 20.3 million were raised for the program (2.5 million per year). These difficulties, coupled with the paradoxical success of DDT, which gave the impression that the disease was nearly under control, led to the gradual collapse of the program and the decision by national health authorities to stop the large-scale use of DDT for malaria control. Finally, the GMEP was officially terminated in 1969, along with the suspension of funding and global control efforts [54].

### 4.4. A Pause in Coordinated Global Efforts to Eradicate Malaria, 1970–1999 

The end of the GMEP coincided with two disastrous decades for the world economy and health. The 1970s were marked by a massive energy crisis in Western economies. Two major oil crises occurred suddenly in 1973 and 1979, when the Yom Kippur War and the Iranian Revolution, respectively, disrupted oil exports from the Middle East [55]. The 1980s were marked by one of the worst public health disasters, the HIV/AIDS pandemic, which attracted the full attention and investment of the world’s health actors [56]. One side effect was the complete disorganization of the global fight against malaria. Gradually, and sometimes after long periods, malaria reappeared in regions where it had been eliminated, returning to high morbidity and mortality rates as the vector population recovered [57,58]. After these two terrible decades, a new global strategy for malaria control was adopted by the World Health Assembly in 1993 [59]. However, this strategy only endorsed the approach considered possible at the time, given the disorganization of member countries’ health systems and the lack of available funding to limit the objective to supporting primary health care in treating patients. For the WHO, this meant relying on decentralized local control programs to strengthen the capacity to diagnose and treat patients, rather than returning to a global approach to parasite and vector control. This strategy worked in some Asian countries, including China, Vietnam, and Thailand, and in South America, particularly Brazil [48]. However, it did not have the desired effect in many countries with historically high levels of transmission. Moreover, the emergence of drug resistance in *P. falciparum* has undermined every new antimalarial treatment over the decades. Indeed, the ability of the parasite to adapt to any type of environmental change or perturbation in its life cycle, such as drug pressure, is a golden rule of survival. Thus, drugs used as monotherapy, those of poor quality, or those administered incompletely or without respect for dosage or duration are likely to generate resistance or escape mechanisms more rapidly by the parasite [60]. Chloroquine, introduced in the 1940s and widely recommended in the 1950s, faced its first instances of resistance in the late 1950s in Colombia and Thailand [61,62]. This resistance later spread to Africa in the 1980s, rendering the drug largely ineffective. Resistance to sulfadoxine-pyrimethamine (SP), recommended in the 1960s as an alternative to chloroquine, emerged in Thailand in the 1960s and spread across Africa during the 1980s and 1990s [61]. Lastly, mefloquine, recommended in the 1980s, encountered resistance in the 1990s, particularly in Southeast Asia, leading to a decline in its use [61,62]. So, during this period, malaria returned to very high levels of transmission, causing several hundred thousand deaths each year, peaking at around 2 million in the late 1990s and early 2000s [63].

### 4.5. The Renewed Global Fight to Eradicate Malaria, from 1998 to Today 

By the end of the twentieth century, despite initial successes, the global health situation in the fight against malaria was deemed a failure. The resources devoted to malaria eradication in the 1950s and 1960s during the GMEP did not achieve their intended results, and subsequent efforts to control the disease also failed to reduce it to acceptable levels. At the beginning of the 21st century, an analysis of the reasons for these failures highlighted several key factors: (i) a lack of coordination of international aid, particularly in the countries most affected by the disease; (ii) an inability to provide and implement malaria control tools on the necessary scale; (iii) inadequate funding for interventions; finally, (iv) a failure to monitor programs and their effects, both positive, such as their effectiveness in reducing morbidity and mortality, and negative, such as the emergence and development of resistance to drugs and insecticides. It was also recognized that eradication is the only viable long-term goal. Therefore, a new, expanded plan was needed, combining a clearly defined strategy, technical objectives with relevant indicators, and sufficient funding to achieve them.

### 4.6. Roll Back Malaria: Definition of the Plan’s Objectives and Technical Implementation, 1998–2015

**Figure 2:**
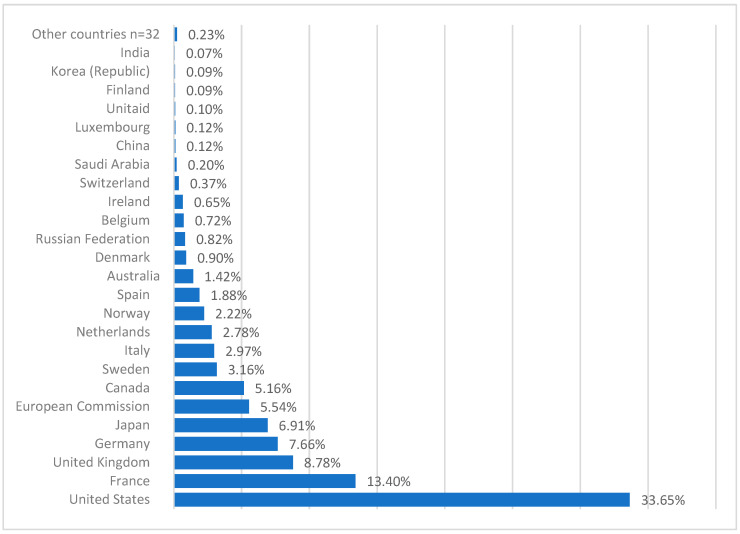
Proportion of the Funding of The Global Fund to Fight AIDS, Tuberculosis, and Malaria, period 2001–2016, public sector. Data source: the Global Fund, https://data.theglobalfund.org/financial-insights (access on 21 August 2024). From 2001 to 2016, a total of USD 40.7 billion was donated to the Global Fund (20% used for malaria), 95% by the public sector, and 5% by the private and nongovernmental sectors.

**Figure 3:**
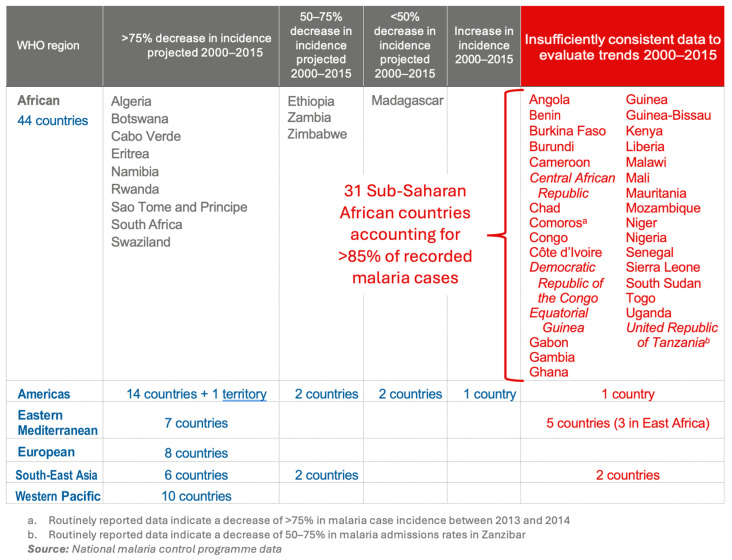
“Table 2.4 Summary of trends in reported malaria case incidence 2000–2015, by WHO region” Modified from World Malaria Report 2015 p14, source WHO, see ref [73].

Under the aegis of the WHO, the necessary elements for structuring this renewed action were gradually put in place. In October 1998, the cornerstone of this new control strategy, the Roll Back Malaria (RBM) Partnership, was launched by the WHO, the World Bank, UNICEF, and the United Nations Development Programme (UNDP). This initiative aimed to halve the burden of malaria by 2010 [64]. It was supported by a global partnership of development agencies, banks, private sector groups (foundations, associations, pharmaceutical companies), and researchers. RBM programs were designed to coordinate global action against malaria, primarily managed by the national authorities of the affected countries. For each specific challenge in the fight against malaria, disease control institutions in endemic countries could draw on networks of resources connecting experts from research institutes and international universities with health teams in the field. Africa was at the heart of this global project. Implementation of the RBM initiative began in 1998 with a series of consultations in participating countries, followed by sub-regional consensus and launch meetings. In 2000, when technical activities commenced, 108 countries were malaria endemic. The estimated number of malaria cases was 245 million, with approximately 860,000 deaths [65,66]. Children under 5 accounted for 66% of these deaths. In Sub-Saharan Africa, this represented 22% of deaths in this age group [67]. In the same year, the United Nations General Assembly adopted a Millennium Development Goal (MDG) on malaria, numbered six on a list of eight. The target, defined in Goal 6C, was to halt and begin to reverse the incidence of malaria by 2015. The indicators chosen to monitor this trend were as follows: 6.6, measuring malaria incidence and mortality; 6.7, the proportion of children under five who sleep under insecticide-treated bed nets; 6.8, the proportion of children under five with fever treated with appropriate antimalarial drugs [64,68]. In 2005, at its 58th World Health Assembly, the WHO reaffirmed in Resolution WHA58.2 the goal of reducing the burden of malaria by at least 50% by 2010 and set a target of a 75% reduction by 2015 [69]. In 2008, the Roll Back Malaria Partnership reaffirmed the previous targets, specifying the goal of “ensuring that the number of preventable deaths is close to zero by 2015” [70]. In just a few years, the WHO, building on experience and mobilizing key partners, was able to re-launch the fight against malaria and develop and implement a new global plan to tackle the disease. This vigorous resumption of the global fight led to a significant reduction in malaria within a few years. After an initial increase in cases and deaths between 2000 and 2003, following the previous trend, the interventions, based on the selected indicators, showed a rapid reduction in cases and in the spatial distribution of malaria worldwide, with a rather spectacular reduction in incidence and mortality rates by 2015 [66]. Looking at the MDG indicators, Indicator 6.6 shows a 37% reduction in the incidence rate and a 60% reduction in the mortality rate by 2015 compared with 2000. In terms of transmission control, Indicator 6.7, the proportion of children under five in Sub-Saharan Africa sleeping under an insecticide-treated bed net, increased from less than 2% in 2000 to around 68% in 2015. In the same region, regular indoor residual spraying with insecticides covered about 7% of the population at risk. Regarding access to appropriate drug treatment for children under five, Indicator 6.8, 13% of febrile children in Sub-Saharan Africa were treated with an ACT in 2015, compared with 0% before 2004 [66,71]. These results raised hopes of achieving the goal of eradicating the disease. This was achieved through significant improvements in planning for action, supported by an expanded and better-coordinated partnership, with increased and stabilized funding over time. Available funding increased six-fold over this period, from around USD 0.5 billion in 2000 to around USD 3 billion per year in 2009. However, in 2015, total funding for malaria was estimated at only USD 2.9 billion, an increase of only USD 0.06 billion since 2010 [66]. In 2015, governments of endemic countries funded 32% of the global fight against malaria. The remaining 68% came from international funding. This funding was provided to endemic countries through bilateral aid or intermediaries such as the Global Fund, the World Bank, and other multilateral institutions. The United States was the largest donor, contributing 35% of the total, followed by the United Kingdom (16%), France (3.2%), Germany (2.4%), and Japan (2.3%). Almost 45% of international funding was channeled through the Global Fund. More than half of international funding went to prevention and treatment. Progress against malaria was therefore highly dependent on fluctuations in international donor spending. It is worth noting the rarely explored political aspect of international commitments in this WHO action. Major countries were only marginally involved. If we look at the sources of funding for the Global Fund for the period 2001–2016, a total of 40.7 billion dollar was donated to the Global Fund (20% for malaria); 95% by the public sector and 5% by the private and nongovernmental sector. Concerning the public sector funding, among the USD 38.6 billion received, the United States contributed 35.23%, France 13.40%, the United Kingdom 8.78%, the Russian Federation 0.82%, and the People’s Republic of China 0.12% (Figure 2). The participation of the Russian Federation and the People’s Republic of China was symbolic. Their involvement was mainly through bilateral actions that are not monitored by the WHO. Unfortunately, global health is not immune to politics. Maintaining a strong influence over the WHO is clearly an issue for the major powers, and this has difficult-to-measure consequences for the coordination and funding of global disease control programs. Other factors include technological innovations such as rapid malaria detection tests, insecticide-treated nets, various ACTs, the development of health systems, and the overall economic development of countries. The most effective control methods were as follows: distribution of insecticide-treated mosquito nets (ITNs), which accounted for 68% (62–72%) of the estimated reduction in prevalence observed in 2015; treatment of infected individuals with effective artemisinin-based treatments (ACTs) and indoor residual spraying (IRS) with insecticides, estimated to have contributed 19% (15–24%) and 13% (11–16%), respectively [72]. These interventions are estimated to have prevented 663 million (542–753 million) cases in 2000–2015, of which 68% (62–73%), 22% (17–28%), and 10% (5–14%) are directly attributable to ITNs, ACTs, and IRS, respectively [72]. However, it should be stressed that these contributions do not reflect the comparative effectiveness of the different intervention strategies, as they also depend on the timeliness and scale of implementation of the different interventions. Despite the generally positive assessment and the enthusiasm shown by the heads of the WHO and UNICEF, not all objectives were achieved. In fact, only the most modest of the targets, that of bringing malaria under control and beginning to reverse the trend, was fully met [71,73]. In absolute terms, the reduction in the burden of malaria was more modest: 8% in cases and 32% in deaths. Thus, even if the trends are not called into question, in 2015, almost half the world’s population, some 3.2 billion people, were still at risk of malaria, and deaths, especially among adults, appear to be seriously underestimated by the WHO [63,67]. Experts at the Institute for Health Metrics and Evaluation in Seattle, using a proven methodology, found that the true number of deaths was 1.3 times higher for children under 5 in Africa, 8.1 times higher for people aged 5 and over in Africa, and 1.8 times higher for people of all ages outside Africa [63]. The other two targets, reducing the burden of malaria by 75% and reducing preventable deaths to near zero, have not been met. Regarding the 75% reduction target, an initial trend analysis based solely on WHO data shows that 31 of the 44 African countries, all in Sub-Saharan Africa, are classified in the column ‘Insufficient consistent data to assess trends 2000–2015’ [73] (Figure 3). Yet, these 31 countries accounted for more than 85% of global malaria cases in 2015 [66]. Fortunately, external data from parasite prevalence surveys conducted in Sub-Saharan Africa in most of the affected countries allowed for the estimation of trends in case incidence.

**Figure 4:**
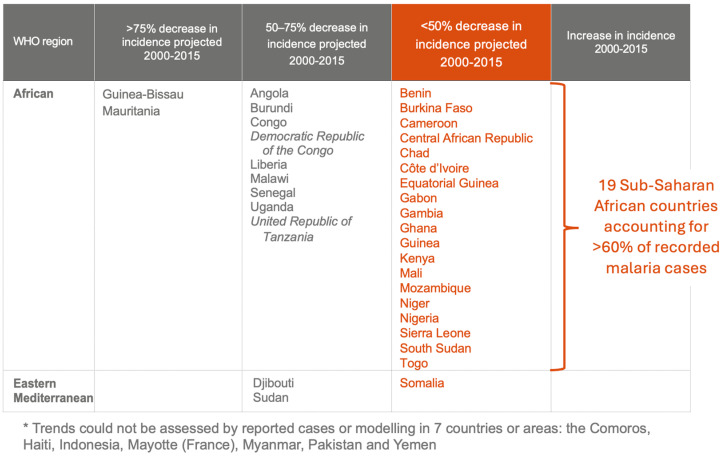
“Table 2.5 Summary of trends in estimated malaria case incidence 2000–2015, for countries in which trends could not be evaluated from reported data but can be assessed through modeling *” Modified from World Malaria Report 2015 p15, source: WHO, see ref [73].

Using this new methodology, the WHO estimated that among 30 of these 31 countries, trends could not be assessed for Comoros, only two countries, Guinea-Bissau and Mauritania, would have met the target of a 75% reduction in incidence, while 19 countries, including Nigeria, did not achieve even a 50% reduction in incidence [73] (Figure 4).

Several factors contribute to this relative failure. The first is the weakness of health systems in the poorest countries, which are often the most affected by the disease and unable to implement effective prevention or care programs. For example, in Sub-Saharan Africa, nearly a third of the population, about 300 million people, live in households without bed nets. Since malaria is concentrated in countries with low national incomes, the cost of malaria treatment is even more burdensome for these economies. Consequently, despite a substantial increase in funding to the WHO, approximately USD 3 billion a year since 2009, with more than 80% allocated to countries in the WHO’s African region, programs have remained underfunded. According to the WHO, only half of planned interventions could be funded during this period [66]. The second is the emergence and spread of *P. falciparum* resistance to artemisinin derivatives. This is a major threat to global malaria control and elimination efforts. ACTs have been the first-line treatment for malaria since the 2000s. However, resistance was first observed in Southeast Asia, notably in Cambodia around 2008 [74]. Since this discovery, resistance has spread to several neighboring countries, including Thailand, Vietnam, and Laos. It is primarily associated with mutations in the kelch13 gene of *P. falciparum* [75]. These mutations delay parasite clearance after treatment, although the effectiveness of ACTs remains partially preserved due to the partner drugs in the combinations. Despite these concerning facts, a new technical plan was adopted and announced by the WHO concurrently with the 2000–2015 plan analysis [71,73].

### 4.7. Global Technical Strategy for Malaria 2016–2030, Including Difficulties, New Challenges, and New Tools

As we discussed in the “Present situation, extent of the disease” chapter, the 2016–2030 Global Technical Strategy for Malaria (GTSM) is failing at its midpoint. Since 2015, the tools that worked until then, i.e., indoor residual spraying of insecticides, treatment of symptomatic cases with artemisinin-based combination therapies (ACTs), and insecticide-treated bed nets, no longer appear to be sufficiently effective, particularly in Sub-Saharan Africa. In the 2010s, the emergence of widespread insecticide resistance became a major challenge for both LLIN and IRS programs [76]. The resistance to pyrethroids led to renewed efforts to develop and deploy new insecticide formulations and combinations. In recent years, non-pyrethroid insecticides, such as the neonicotinoid clothianidin and the pyrrole chlorfenapyr, have been introduced for IRS in areas with high levels of resistance [77]. The Global Plan for Insecticide Resistance Management (GPIRM), launched by the WHO in 2012, has been critical in guiding countries in managing resistance through rotating insecticides and integrating other vector control methods [78]. However, despite these efforts, IRS coverage has been declining globally due to operational challenges, funding constraints, and the logistical complexity of implementing IRS on a large scale. By 2022, only 47 out of 85 countries were using IRS to prevent malaria. Overall, the proportion of the at-risk population covered by IRS has fallen from 5.5% in 2010 to 1.8% in 2022 in these countries and from 5.2% to just 0.7% in the countries of the WHO African region. [6]. In Africa, where the malaria burden is highest, the use of ACTs in children under 5 years of age increased from a median of 13% at baseline (2005–2011 surveys) to 24% in the most recent survey (2015–2022 surveys) [6]. This means that three out of four children were not receiving the recommended therapy. Worst of all, signs of artemisinin resistance began to appear in 2015–2017, particularly in Uganda, Rwanda, and more recently in Kenya and Ethiopia [79,80,81,82].

Another major concern is the recent invasion and expansion of *Anopheles stephensi* in Africa. *Anopheles stephensi* is a mosquito species traditionally found in South Asia, the Middle East, and parts of the Arabian Peninsula, where it is a major vector of urban and peri-urban malaria. It is well adapted to man-made environments, often breeding in water storage tanks, wells, and other artificial water bodies [83]. It can rest both indoors and outdoors but also exploit both animal and human hosts. This is likely to reduce the impact of IRS and LLINs. Moreover, this mosquito species is also known for its resistance to common insecticides, complicating vector control efforts [83]. *Anopheles stephensi* was first detected in Africa in 2012 in Djibouti [84]. The Republic of Djibouti is a semi-arid country in the Horn of Africa. It has a population of about 900,000, 70% of whom live in the capital, Djibouti City. Prior to 2013, malaria was hypoendemic, with low levels of transmission only in peri-urban and rural areas. In 2013, a malaria outbreak occurred in Djibouti City, and entomological studies identified *A. stephensi* mosquitoes as a new malaria vector. In 2018, malaria diagnosis increased to 25,319 confirmed cases in the capital; more than 100,000 were suspected, approximately 64% *P. falciparum* and 36% *P. vivax* [85]. By 2016, *A. stephensi* was reported in Ethiopia, particularly in urban areas such as Dire Dawa [86]. In the years following its first detection in Dire Dawa City, a 12-fold increase in malaria incidence, predominantly *P. falciparum*, was observed in the city following a malaria outbreak in April–July 2022. Worryingly, this outbreak involved the clonal propagation of parasites with molecular signatures of artemisinin and diagnostic resistance [87]. Between 2019 and 2022, it continued to spread in East Africa, with confirmed reports in Sudan, Somalia, and recently, in northern Kenya [83,88]. Most worryingly but yet to be confirmed, it is now reported in two major cities in West Africa, Nigeria and Ghana [6,83,89]. Its ability to thrive in urban environments, coupled with the increasing urbanization of SSA, poses a significant risk of increased malaria transmission in cities that were previously considered lower-risk areas. It should be noted that the population living in urban areas in Africa has increased from 31.5% in 1990 to 42.5% in 2018 and that about 60% of the population is expected to live in urban areas by 2050 [90]. In various press releases starting in 2019, then in its annual reports in 2021, 2022, and 2023, and finally, in a special publication in 2023, the WHO raised concerns about the spread of *A. stephensi* in Africa, urging countries to strengthen surveillance and implement specific control measures [6,91,92].

In addition, a new pandemic has had a major negative impact on the course of malaria control efforts. The COVID-19 pandemic has severely disrupted local health systems in 21 of the 48 countries most affected by the epidemic. It is estimated to be responsible for an additional 14 million malaria cases and 90,000 malaria deaths in 2020 compared to 2019 [9]. On a smaller scale, political and economic crises exacerbate the situation. Venezuela, which managed to control the disease in the early 2000s, provides a particularly instructive example. Since the mid-2000s, Venezuela has faced a severe economic crisis caused by political instability and declining oil revenues. Public health services have been particularly hard hit. As this crisis has worsened, the country has seen an alarming increase in malaria cases, from about 45,000 cases in 2010 to about 136,000 in 2015 and about 470,000 in 2020, a 1200% increase compared with the year 2000, making Venezuela one of the most malaria-affected countries in the Americas [9,93].

Few solutions appear to be rapidly applicable to reverse this trend. The WHO has launched several robust initiatives. The first, launched in 2018, called High Burden to High Impact (HBHI), targeted the 11 countries most affected by malaria, Burkina Faso, Cameroon, Democratic Republic of the Congo, Ghana, India, Mali, Mozambique, Niger, Nigeria, Uganda, and the United Republic of Tanzania, to implement enhanced control interventions adapted to the local context. These efforts did not pay off, particularly during the COVID-19 crisis, when all HBHI countries except India reported an increase in cases and deaths between 2019 and 2020 [9]. Overall, malaria cases in these countries increased from 154 million cases and 398,000 deaths in 2019 to 163 million cases and 444,600 deaths in 2020 [9]. The results were no better in 2021 and 2022 [6,94]. Against this backdrop, in late 2021, the WHO recommended the widespread use of a malaria vaccine against *P. falciparum* RTS,S/AS01 in children in Sub-Saharan Africa and other regions with moderate to high transmission of *P. falciparum* malaria [95,96]. RTS,S is a fusion protein of the hepatitis B surface antigen and the particle containing the carboxy-terminal and 18 NANP copies of the highly repetitive region of the *P. falciparum* circumsporozoite protein (PfCSP). PfCSP is the dominant surface antigen of the sporozoite stage of the parasite injected by the insect vector to invade its human host and is critical for parasite invasion of liver cells. Development of the RTS,S/AS01 vaccine took approximately thirty years. It is well tolerated, but vaccine efficacy is limited, at less than 40% four years after a four-injection schedule in young children [97]. This first action was quickly followed by a new recommendation in October 2023 for a vaccine candidate, R21/Matrix-M, developed by the University of Oxford (UK) [95]. The R21/Matrix-M vaccine is well tolerated and has demonstrated a superior vaccine efficacy of 77% at one year [98]. The target is the same, the RTS,S fusion protein, but what changes is the higher amount of parasite protein incorporated into the vaccine particle and, most importantly, the efficacy of the adjuvant, which impacts its protection and probably its duration. Other vaccine solutions are expected, particularly those using mRNA technology [99,100]. The widespread use of the vaccine strategy is justified by the current poor control results and the lack of alternative solutions that can be rapidly deployed on a global scale. Estimates derived from mathematical modeling indicate that this vaccine strategy should have a strong public health impact in a wide range of malaria transmission settings, even where transmission is low [101].

In addition to the development of new malaria vaccines, there have been recent R&D advances in the development of monoclonal antibodies (mAbs) for the prevention of malaria. Monoclonal antibodies are laboratory-produced molecules that can mimic the immune system’s ability to fight off harmful pathogens (viruses, bacteria, yeasts and parasites). So, passive immunization with mAbs, which involves directly administering functional antibodies, could overcome vaccine limitations by providing immediate protection [102]. Recent clinical trials of anti-invasion or transmission-blocking mAbs against *P. falciparum* have shown promising results. Among the most promising are the subcutaneously or intravenously injected mAbs CIS43LS and L9LS, which both target the PfCSP and have demonstrated significant protective efficacy against malaria for up to 9 months after administration [103,104,105]. This represents a promising approach in the fight against malaria, offering a potential complementary strategy to existing vaccines and treatments, particularly in areas where drug resistance is a problem [99,100].

## 5. Conclusions

The global fight against malaria has been an uphill battle for more than a century. The WHO’s coordination of the fight since 1948 led to significant progress and high hopes, particularly between 2004 and 2015. But, the WHO’s technical plan for 2016–2030 has failed to meet its milestone targets. In fact, there is a good chance that this plan will fail to achieve any of its stated goals by the deadline. It is important to note that Sub-Saharan Africa is particularly affected. It now accounts for more than 90% of the world’s cases and deaths. So, to revive the fight, it is now imperative to better understand the challenges specific to this region. The epidemiology of the disease in Africa is complicated by a complex interplay of environmental, socio-economic, political and structural factors, particularly in the health sector. These peculiarities are not fully understood, but they have often made past and present malaria control efforts less effective. Added to this is the growing global problem of resistance of mosquito vectors to insecticides and of the malaria parasite to key antimalarial drugs such as artemisinin. This resistance requires constant monitoring and the urgent development of new drugs, alternative tools, and new strategies to maintain the effectiveness of the fight and prevent the disease from resurfacing in regions and areas weakened by often brutal health, climatic, political, or social events. Given the challenges facing Africa, it is essential to develop tailored strategies that address the specific needs and constraints of countries in the region. Some solutions are already known and could be implemented in the short term. For example, it is imperative to strengthen monitoring and evaluation mechanisms that provide accurate, real-time data on the effectiveness of programmed interventions. Strengthening these systems will not only improve the responsiveness of malaria control programs but also quickly identify areas where current strategies are falling short. The development of new tools, such as vaccines, monoclonal antibodies, and new insecticide formulations, as well as innovative delivery methods, such as the combination of indoor residual spraying and long-lasting insecticide-treated nets, are seen as essential steps to sustain the impact of malaria control efforts. It is now essential to invest heavily in these innovative approaches and to test and adapt existing interventions to local contexts. By focusing on these areas, the global health community can better help Sub-Saharan Africa overcome the barriers that have hindered progress in the past and ensure that the most affected regions are not left behind in the global fight against this devastating disease.

## Data Availability

Not applicable.

## References

[1] White N.J., Pukrittayakamee S., Hien T.T., Faiz M.A., Mokuolu O.A., Dondorp A.M. (2014). Malaria. Lancet.

[2] Snounou G., Sharp P.M., Culleton R. (2024). The two parasite species formerly known as *Plasmodium ovale*. Trends Parasitol..

[3] Sutherland C.J., Tanomsing N., Nolder D., Oguike M., Jennison C., Pukrittayakamee S., Dolecek C., Hien T.T., do Rosário V.E., Arez A.P. (2010). Two nonrecombining sympatric forms of the human malaria parasite *Plasmodium ovale* occur globally. J. Infect. Dis..

[4] Carter R., Mendis K.N. (2002). Evolutionary and historical aspects of the burden of malaria. Clin. Microbiol. Rev..

[5] Causes of Death. Our World in Data. https://ourworldindata.org/grapher/annual-number-of-deaths-by-cause.

[6] (2023). World Malaria Report 2023.

[7] Lambert M.-L., van der Stuyft P. (2002). Editorial: Global Health Fund or Global Fund to fight AIDS, Tuberculosis, and Malaria?. Trop. Med. Int. Health.

[8] (2021). Global Technical Strategy for Malaria 2016–2030, 2021 Update. https://www.who.int/publications/i/item/9789240031357.

[9] (2021). World Malaria Report 2021. https://www.who.int/publications/i/item/9789240040496.

[10] Shampo M.A., Kyle R.A. (1989). Nei Ching—Oldest Known Medical Book. Mayo Clin. Proc..

[11] Adams (Francis) (1843). The Genuine Works of Hippocrates. Of the Epidemics 1.6,7,24–26; Aphorisms 3.21,22;4.59,63; on Airs, Waters and Places c. 10. https://classicalliberalarts.com/resources/HIPPOCRATES_1.pdf.

[12] Arrow K.J., Panosian C., Gelband H. (2004). A Brief History of Malaria. Saving Lives, Buying Time: Economics of Malaria Drugs in an Age of Resistance.

[13] Ambroise-Thomas P. (2007). *La petite et la grande histoire du paludisme* (The short and long history of malaria). Bull. Acad. Natle Méd..

[14] Michel M., Skourtanioti E., Pierini F., Guevara E.K., Mötsch A., Kocher A., Barquera R., Bianco R.A., Carlhoff S., Coppola Bove L. (2024). Ancient *Plasmodium* genomes shed light on the history of human malaria. Nature.

[15] Thompson T. (2024). Ancient malaria genome from Roman skeleton hints at disease’s history. Nature.

[16] Hawass Z., Gad Y.Z., Ismail S., Khairat R., Fathalla D., Hasan N., Ahmed A., Elleithy H., Ball M., Gaballah F. (2010). Ancestry and pathology in King Tutankhamun’s family. JAMA.

[17] Nerlich A.G., Schraut B., Dittrich S., Jelinek T., Zink A.R. (2008). *Plasmodium falciparum* in ancient Egypt. Emerg. Infect. Dis..

[18] Mitchell P.D. (2024). Parasites in ancient Egypt and Nubia: Malaria, schistosomiasis and the pharaohs. Adv. Parasitol..

[19] Boualam M.A., Heitzmann A., Mousset F., Aboudharam G., Drancourt M., Pradines B. (2023). Use of rapid diagnostic tests for the detection of ancient malaria infections in dental pulp from the sixth century in Versailles, France. Malar. J..

[20] Laveran A. (1884). Traité des Fièvres Palustres.

[21] Buisson Y. (2023). *Laveran et l’Académie nationale de médecine* [Laveran and the French Academy of Medicine]. Med. Trop. Sante Int..

[22] Ross R. (1897). On some Peculiar Pigmented Cells Found in Two Mosquitos Fed on Malarial Blood. BMJ.

[23] Grassi B., Bignami A., Bastianelli G. (1899). Medical Zoology: Further Researches upon the Cycle of Human Malaria in the Body of the Mosquito. Ind. Med. Gaz..

[24] (2022). How Malaria Shaped Our History “Comment le paludisme a façonné notre histoire” with Pr Renaud Piarroux. Mecaniques des Epidemies Saison 5: Le Paludisme. Paris. https://www.radiofrance.fr/franceculture/podcasts/mecaniques-des-epidemies/comment-le-paludisme-a-faconne-notre-histoire-3885057.

[25] Roersch van der Hoogte A., Pieters T. (2014). Science in the service of colonial agro-industrialism: The case of cinchona cultivation in the Dutch and British East Indies, 1852–1900. Stud. Hist. Philos. Sci. Part C.

[26] Greenwood D. (1992). The quinine connection. J. Antimicrob. Chemother..

[27] (1958). The First Ten Years of the World Health Organization.

[28] Hay S.I., Guerra C.A., Tatem A.J., Noor A.M., Snow R.W. (2004). The global distribution and population at risk of malaria: Past, present, and future. Lancet Infect. Dis..

[29] Chen T.T., Ljungqvist F.C., Castenbrandt H., Hildebrandt F., Ingholt M.M., Hesson J.C., Ankarklev J., Seftigen K., Linderholm H.W. (2021). The spatiotemporal distribution of historical malaria cases in Sweden: A climatic perspective. Malar. J..

[30] Najera J.A. (1989). Malaria and the work of WHO. Bull. World Health Organ..

[31] Litsios S. (1997). Malaria control, the cold war, and the postwar reorganization of international assistance. Med. Anthropol..

[32] Mertens J.E. (2024). A History of Malaria and Conflict. Parasitol. Res..

[33] Amelineau F. (1992). Désastre sanitaire. Rev. Hist. Des Armées.

[34] Richard-Lenoble D., Danis M., Saliou P. (2013). *La médecine tropicale d’hier à aujourd’hui* [Tropical medicine: Past and present]. Bull. Acad. Natl. Med..

[35] Faust E.C. (1951). The History of Malaria in the United States. Am. Sci..

[36] Prinzi A., Rohde R. (2023). The History of Malaria in the United States. https://asm.org/Articles/2023/September/The-History-of-Malaria-in-the-United-States.

[37] Carter E.D. (2014). Malaria control in the Tennessee Valley Authority: Health, ecology, and metanarratives of development. J. Hist. Geogr..

[38] (1996). Office of the Director, Epidemiology Program Office, CDC Historical Perspectives History of CDC. MMWR.

[39] The Editors of Encyclopaedia Britannica (2024). Industrial Revolution. https://www.britannica.com/event/Industrial-Revolution.

[40] Piperaki E.-T., Manguin S., Dev V. (2018). Malaria Eradication in the European World: Historical Perspective and Imminent Threats. Towards Malaria Elimination.

[41] Manguin S., Carnevale P., Mouchet J., Coosemans M., Julvez J., Richard-Lenoble D., Sircoulon J. (2008). Biodiversity of Malaria in the World.

[42] Najera J.A. (1990). Malaria control: Present situation and need for historical research. Parassitologia.

[43] Packard R.M. (1997). Malaria dreams: Postwar visions of health and development in the Third World. Med. Anthropol..

[44] Nájera J.A., González-Silva M., Alonso P.L. (2011). Some lessons for the future from the Global Malaria Eradication Programme (1955–1969). PLoS Med..

[45] Tognotti E. (2009). Program to eradicate malaria in Sardinia, 1946–1950. Emerg. Infect. Dis..

[46] Andrews J.M., Grant J.S., Fritz R.F. (1954). Effects of suspended residual spraying and of imported malaria on malaria control in the USA. Bull. World Health Organ..

[47] Cavalié P., Mouchet J. (1961). La Campagne Expérimentale d’Eradication du Paludisme dans le Nord de la République du Cameroun.

[48] Trigg P.I., Kondrachine A.V. (1998). Commentary: Malaria control in the 1990s. Bull. World Health Organ..

[49] Nájera J.A. (2001). Malaria control: Achievements, problems and strategies. Parassitologia.

[50] Mulliken D.L., Zambone J.D., Rolph C.G. (2005). DDT: A Persistent Lifesaver. Nat. Resour. Environ..

[51] Carter R. (1999). The World Health Report 1999: Making a Difference, Chapter 4 Rolling Back Malaria, Box 4.1.

[52] Beard J. (2006). DDT and human health. Sci. Total Environ..

[53] Roberts D.R., Tren R. (2011). International advocacy against DDT and other public health insecticides for malaria control. Res. Rep. Trop. Med..

[54] (2020). Malaria Eradication: Benefits, Future Scenarios and Feasibility.

[55] Kettell S. (2024). Oil Crisis. The Editors of Encyclopedia Britannica. Britannica. https://www.britannica.com/money/oil-crisis.

[56] Shilts R. (1987). And the Band Played on: Politics, People, and the AIDS Epidemic.

[57] (1999). Rolling back malaria. TDR News.

[58] Najera J.A. (1999). Global Partnership to Roll Back Malaria. Malaria Control: Achievements, Problems and Strategies.

[59] World Health Assembly 46 (1993). Forty-Sixth World Health Assembly, Geneva, 3–14 May 1993: Resolutions and Decisions, Annexes.

[60] White N.J. (2004). Antimalarial drug resistance. J. Clin. Investig..

[61] Pradines B., Dormoi J., Briolant S., Bogreau H., Rogier C. (2010). La résistance aux antipaludiques. Rev. Francoph. Des Lab..

[62] Farooq U., Mahajan R.C. (2004). Drug resistance in malaria. J. Vector Borne Dis..

[63] Murray C.J.L., Rosenfeld L.C., Lim S.S., Andrews K.G., Foreman K.J., Haring D., Fullman N., Naghavi M., Lozano R., Lopez A.D. (2012). Global malaria mortality between 1980 and 2010: A systematic analysis. Lancet.

[64] World Health Organization (2003). Global Partnership to Roll Back Malaria; World Bank; United Nations Development Programme; African Summit on Roll Back Marlaria (2000: Abuja, N.; United Nations Children’s Fund (UNICEF). The Abuja Declaration and the Plan of Action: An Extract from the African Summit on Roll Back Marlaria, Abuja, 25 April 2000 (WHO/CDS/RBM/2000.17). https://iris.who.int/handle/10665/67816.

[65] Roser M., Ritchie H. (2024). Malaria. Our World in Data. https://ourworldindata.org/malaria.

[66] (2016). World Malaria Report 2016.

[67] (2016). WHO/UNICEF report: Malaria MDG target achieved a mid-sharp drop in cases and mortality, but 3 billion people remain at risk. Neurosciences.

[68] (2000). United Nations Millennium Declaration: General Assembly Resolution 55/2, Chap III Development and Poverty Eradication, Num. 19. United Nations, New York. https://www.ohchr.org/en/instruments-mechanisms/instruments/united-nations-millennium-declaration.

[69] World Health Assembly 58 (2005). Fifty-Eighth World Health Assembly, Geneva, 16–25 May 2005: Resolutions and Decisions: Annex.

[70] (2008). The Global Malaria Action Plan For a Malaria Free World.

[71] (2015). Achieving the Malaria MDG Target: Reversing the Incidence of Malaria 2000–2015.

[72] Bhatt S., Weiss D.J., Cameron E., Bisanzio D., Mappin B., Dalrymple U., Battle K., Moyes C.L., Henry A., Eckhoff P.A. (2015). The effect of malaria control on *Plasmodium falciparum* in Africa between 2000 and 2015. Nature.

[73] (2015). World Malaria Report 2015.

[74] Dondorp A.M., Nosten F., Yi P., Das D., Phyo A.P., Tarning J., Lwin K.M., Ariey F., Hanpithakpong W., Lee S.J. (2009). Artemisinin resistance in *Plasmodium falciparum* malaria. N. Engl. J. Med..

[75] Ariey F., Witkowski B., Amaratunga C., Beghain J., Langlois A.-C., Khim N., Kim S., Duru V., Bouchier C., Ma L. (2014). A molecular marker of artemisinin-resistant *Plasmodium falciparum* malaria. Nature.

[76] Ranson H., Lissenden N. (2016). Insecticide Resistance in African Anopheles Mosquitoes: A Worsening Situation that Needs Urgent Action to Maintain Malaria Control. Trends Parasitol..

[77] Thiomela R.F., Tchouakui M., Menze B.D., Nchoutpouen E., Ngongang-Yipmo E.S., Wood O., Horstmann S., Mahob R.J., Fomena A., Wondji C.S. (2024). Indoor residual spraying of experimental huts in Cameroon highlights the potential of Fludora^®^ Fusion to control wild pyrethroid-resistant malaria vectors. BMC Infect. Dis..

[78] (2012). Global Plan for Insecticide Resistance Management in Malaria Vectors: Executive Summary.

[79] Dhorda M., Kaneko A., Komatsu R., Kc A., Mshamu S., Gesase S., Kapologwe N., Assefa A., Opigo J., Adoke Y. (2024). Artemisinin-resistant malaria in Africa demands urgent action. Science.

[80] Uwimana A., Legrand E., Stokes B.H., Ndikumana J.-L.M., Warsame M., Umulisa N., Ngamije D., Munyaneza T., Mazarati J.-B., Munguti K. (2020). Emergence and clonal expansion of in vitro artemisinin-resistant *Plasmodium falciparum* kelch13 R561H mutant parasites in Rwanda. Nat. Med..

[81] Conrad M.D., Asua V., Garg S., Giesbrecht D., Niaré K., Smith S., Namuganga J.F., Katairo T., Legac J., Crudale R.M. (2023). Evolution of Partial Resistance to Artemisinins in Malaria Parasites in Uganda. N. Engl. J. Med..

[82] Jeang B., Zhong D., Lee M.-C., Atieli H., Yewhalaw D., Yan G. (2024). Molecular surveillance of Kelch 13 polymorphisms in *Plasmodium falciparum* isolates from Kenya and Ethiopia. Malar. J..

[83] Taylor R., Messenger L.A., Abeku T.A., Clarke S.E., Yadav R.S., Lines J. (2024). Invasive Anopheles stephensi in Africa: Insights from Asia. Trends Parasitol..

[84] Faulde M.K., Rueda L.M., Khaireh B.A. (2014). First record of the Asian malaria vector *Anopheles stephensi* and its possible role in the resurgence of malaria in Djibouti, Horn of Africa. Acta Trop..

[85] de Santi V.P., Khaireh B.A., Chiniard T., Pradines B., Taudon N., Larréché S., Mohamed A.B., de Laval F., Berger F., Gala F. (2021). Role of *Anopheles stephensi* Mosquitoes in Malaria Outbreak, Djibouti, 2019. Emerg. Infect. Dis..

[86] Carter T.E., Yared S., Gebresilassie A., Bonnell V., Damodaran L., Lopez K., Ibrahim M., Mohammed S., Janies D. (2018). First detection of *Anopheles stephensi* Liston, 1901 (*Diptera: Culicidae*) in Ethiopia using molecular and morphological approaches. Acta Trop..

[87] Emiru T., Getachew D., Murphy M., Sedda L., Ejigu L.A., Bulto M.G., Byrne I., Demisse M., Abdo M., Chali W. (2023). Evidence for a role of *Anopheles stephensi* in the spread of drug- and diagnosis-resistant malaria in Africa. Nat. Med..

[88] Ochomo E.O., Milanoi S., Abong’o B., Onyango B., Muchoki M., Omoke D., Olanga E., Njoroge L., Juma E.O., Otieno J.D. (2023). Detection of *Anopheles stephensi* Mosquitoes by Molecular Surveillance, Kenya. Emerg. Infect. Dis..

[89] Afrane Y.A., Abdulai A., Mohammed A.R., Akuamoah-Boateng Y., Owusu-Asenso C.M., Sraku I.K., Yanney S.A., Malm K., Lobo N.F. (2023). First detection of *Anopheles stephensi* in Ghana using molecular surveillance. Biorxiv.

[90] (2019). World Urbanization Prospects: The 2018 Revision.

[91] (2019). Vector Alert: Anopheles Stephensi Invasion and Spread: Horn of Africa, the Republic of the Sudan and Surrounding Geographical Areas, and Sri Lanka: Information Note.

[92] (2023). Vector Alert: Anopheles Stephensi Invasion and Spread in Africa and Sri Lanka.

[93] Grillet M.E., Villegas L., Oletta J.F., Tami A., Conn J.E. (2018). Malaria in Venezuela requires response. Science.

[94] (2022). World Malaria Report 2022.

[95] WHO (2024). Malaria vaccine: WHO position paper—May 2024. WER.

[96] Lancet T. (2021). Malaria vaccine approval: A step change for global health. Lancet.

[97] (2015). Efficacy and safety of RTS,S/AS01 malaria vaccine with or without a booster dose in infants and children in Africa: Final results of a phase 3, individually randomised, controlled trial. Lancet.

[98] Datoo M.S., Dicko A., Tinto H., Ouédraogo J.-B., Hamaluba M., Olotu A., Beaumont E., Ramos Lopez F., Natama H.M., Weston S. (2024). Safety and efficacy of malaria vaccine candidate R21/Matrix-M in African children: A multicentre, double-blind, randomised, phase 3 trial. Lancet.

[99] Laurens M.B. (2021). Novel malaria vaccines. Hum. Vaccin. Immunother..

[100] Miura K., Flores-Garcia Y., Long C.A., Zavala F. (2024). Vaccines and monoclonal antibodies: New tools for malaria control. Clin. Microbiol. Rev..

[101] Schmit N., Topazian H.M., Natama H.M., Bellamy D., Traoré O., Somé M.A., Rouamba T., Tahita M.C., Bonko M.D.A., Sourabié A. (2024). The public health impact and cost-effectiveness of the R21/Matrix-M malaria vaccine: A mathematical modelling study. Lancet Infect. Dis..

[102] (2023). Monoclonal Antibodies for Malaria Prevention: Preferred Product Characteristics and Clinical Development Considerations.

[103] Gaudinski M.R., Berkowitz N.M., Idris A.H., Coates E.E., Holman L.A., Mendoza F., Gordon I.J., Plummer S.H., Trofymenko O., Hu Z. (2021). A Monoclonal Antibody for Malaria Prevention. N. Engl. J. Med..

[104] Lyke K.E., Berry A.A., Mason K., Idris A.H., O’Callahan M., Happe M., Strom L., Berkowitz N.M., Guech M., Hu Z. (2023). Low-dose intravenous and subcutaneous CIS43LS monoclonal antibody for protection against malaria (VRC 612 Part C): A phase 1, adaptive trial. Lancet Infect. Dis..

[105] Wu R.L., Idris A.H., Berkowitz N.M., Happe M., Gaudinski M.R., Buettner C., Strom L., Awan S.F., Holman L.A., Mendoza F. (2022). Low-Dose Subcutaneous or Intravenous Monoclonal Antibody to Prevent Malaria. N. Engl. J. Med..

